# An evaluation of osmolality measurement by freezing point depression using micro-amounts of sample

**DOI:** 10.1155/S1463924689000167

**Published:** 1989

**Authors:** G. Koumantakis, L. E. Wyndham

**Affiliations:** Biochemistry Department Royal North Shore Hospital of Sydney Sydney 2065 Australia

## Abstract

An evaluation of the Advanced micro-osmometer is presented. This instrument has been shown to have an excellent analytical precision (within-run CV = 0.59%, between-day CV = 0.58%). It is accurate over an analytical range of 0-2000 mmol/kg of osmolality shown by linearity studies and split sample correlations against vapour pressure osmometry, freezing point osmometry and an external quality assurance programme. Analytical errors due to operator technique are almost eliminated because of good instrument design. Preliminary results on whole-blood osmolality are included. The required sample size of 20 μl permits osmolality measurements on most clinical samples. It is concluded that the Advanced micro-osmometer satisfies laboratory requirements.


Journal of Automatic Chemistry Vol. 11, No. 2 (March–April 1989), pp. 80–83

### An evaluation of osmolality measurement by freezing point depression using micro-amounts of sample

**G. Koumantakis and L. E. Wyndham**

*Biochemistry Department, Royal North Shore Hospital of Sydney, Sydney, 2065, Australia*

*An evaluation of the Advanced micro-osmometer is presented. This instrument has been shown to have an excellent analytical precision (within-run CV = 0·59%, between-day CV = 0·58%). It is accurate over an analytical range of 0–2000 mmol/kg of osmolality shown by linearity studies and split sample correlations against vapour pressure osmometry, freezing point osmometry and an external quality assurance programme. Analytical errors due to operator technique are almost eliminated because of good instrument design. Preliminary results on whole-blood osmolality are included. The required sample size of 20 μl permits osmolality measurements on most clinical samples. It is concluded that the Advanced micro-osmometer satisfies laboratory requirements.*

### Introduction

Changes in departmental requirements concerning the measurement of fluid osmolality initiated this study. Our laboratory is currently equipped with instrumentation that utilizes the principle of vapour pressure osmometry but, because of the poor performance of this procedure in our external quality assurance programme, it was decided to investigate the osmolality of solutions by measurement of freezing point depression, as this method has been shown to perform well in such programmes.

Freezing point in osmometry is that temperature (at atmospheric pressure) at which the solid and liquid phases will co-exist in equilibrium. When a solute is dissolved in a pure solvent, the colligative properties of the solvent usually change in direct proportion to the solute concentration. Measurement of the freezing point allows concentrations to be determined with greatest precision owing to the inherent isolation of the sample from the environment by the iced blanket generated when the sample freezes.

Apart from the analytical principle requirement, sample size was an important consideration. We perform sweat osmolalities for the detection of cystic fibrosis as described by Webster and co-workers [1–3] using a collection procedure described by Carter *et al.* [4] and receive paediatric samples and body fluids not easily obtained in large amounts. Primary selection of instruments was based on impressions gained from commercial advertising and it was decided to assess the performance of the Model 3MO Advanced micro-osmometer (Advanced Instruments, Needham Heights, MA, USA).

### Experimental

The Advanced micro-osmometer was supplied by Stansens Scientific (Sydney, Australia) for this study. This instrument uses 20 μl of sample to measure sample osmolality by freezing point depression. Supercooling of the sample is initiated by the insertion of a specially designed, disposable sample holder containing the sample into the instrument's thermistor probe, which is in a fixed position. Following a solenoid-induced pulse and subsequent sample freezing, the liberated heat of fusion is related by a microprocessor to the sample's freezing point and osmolality is shown on a digital display. Calibration of the instrument requires no adjustment by the operator. It consists of running 2–6 samples at each of two calibration levels (50 and 850 mmol/kg). If the repeatability is acceptable, the instrument automatically performs internal calibration.

### *Samples*

*Within-run precision.* Five types of sample were used in order to evaluate the repeatability of the instrument. Aqueous, commercially available solutions of 50, 290 and 850 mmol/kg (Advanced Instruments, P.N. 3MA005, Clinitrol 290 and 3MA085, respectively), plasma obtained by centrifugation from lithium heparinized whole blood, whole blood, three 24-h urine samples collected with 5 g of thymol and pooled eccrine sweat collected from volunteers by pilocarpine iontophoresis using the Webster Model 3600 sweat inducer and microduct kit (Webster Scientific, Sydney, Australia). Each sample was analysed ten times (except the sweat sample, which was analysed five times) in a single batch and on the same day. The osmolalities measured were selected to span the instrument's analytical range of 0–2000 mmol/kg.

*Between-day precision.* To avoid sample evaporation and subsequent changes in sample osmolality, samples selected for this part of the study were refrigerated throughout and an effort was made to exclude as much air as possible from the sample tube. Prior to each analysis, aliquots were brought to room temperature and analysed in random order amongst a run of other specimens. Samples were selected from patients and quality control material, with appropriate osmolality levels, and analysed in duplicate on nine separate runs over a period of eight days.

*Accuracy studies.* Ninety-four samples were selected from patients' plasma and urine specimens analysed by the

0142–0453/89 $3.00 © 1989 Taylor & Francis Ltd.

Wescor vapour pressure osmometer so that the levels of osmolality were distributed throughout the analytical range. Haemolysed, lipaemic and icteric specimens were also included. These samples were analysed in duplicate with the micro-osmometer. In addition, the accuracy of the micro-osmometer was also investigated by analysing 25 urine and plasma samples with both the Advanced micro-osmometer and a Knauer semi-micro osmometer (Knauer, Berlin, FRG), which also utilizes freezing point depression thermodynamics. In addition, 11 lyophilized samples with target values previously assigned and linearly related were obtained from the Royal College of Pathologists of Australasia Quality Assurance Programme (Target Kit No. 13, Samples 1–11), reconstituted and analysed in duplicate with the Advanced micro-osmometer.

*Linearity studies.* A urine sample with an osmolality of 1918 mmol/kg was selected and 0-, 0·2-, 0·4-, 0·6-, 0·8- and 1·0-ml aliquots were pipetted into test-tubes in duplicate. Into each of the aliquots 1·0, 0·8, 0·6, 0·4, 0·2 and 0 ml of distilled water were added in sequence, resulting in six diluted samples with calculated osmolalities of 0, 384, 767, 1150, 1534 and 1918 mmol/kg, respectively. Both sets of aliquots were analysed in duplicate with the micro-osmometer in two different analytical batches.

*Whole-blood osmolality.* Thirty-four heparinized samples submitted to the laboratory for routine biochemical analysis were used to measure whole-blood osmolality. Each sample was analysed for osmolality and after centrifugation the plasma osmolality was measured.

*Between-operator variation.* Because of the small amount of sample required to perform each analysis on the micro-osmometer, we decided to measure the instrument's dependence on operator technique. Seven members of the laboratory staff were selected from five technical and professional types. Each was instructed on the basic operation of the instrument and was then given a sample to analyse ten times.

### Results

*Within-run precision*

The results are shown in table 1. The samples selected spanned an analytical range of 37–1917 mmol/kg, sufficient to cover expected results most often submitted to our laboratory for routine analysis. Acceptable standard deviations (SD) and coefficients of variations (CV) of 0·17–1·30% (average 0·59%) were obtained.

*Table 1. Within-run precision*

| No. of analyses | Mean (mmol/kg) | SD (mmol/kg) | CV (%) | Sample type |
|---|---|---|---|---|
| 10 | 36·7 | 0·49 | 1·30 | Urine |
| 10 | 49·8 | 0·42 | 0·85 | Aqueous |
| 5 | 124·0 | 0·92 | 0·74 | Eccrine sweat |
| 10 | 289·3 | 0·48 | 0·17 | Aqueous |
| 10 | 293·4 | 2·98 | 1·02 | Whole blood |
| 10 | 295·6 | 1·00 | 0·35 | Plasma |
| 10 | 557·6 | 0·97 | 0·17 | Urine |
| 10 | 852·4 | 1·76 | 0·21 | Aqueous |
| 10 | 1917·6 | 9·20 | 0·48 | Urine |

*Table 2. Between-day precision*

| No. of runs | Target | Mean (mmol/kg) | SD (mmol/kg) | CV (%) | Sample type |
|---|---|---|---|---|---|
| 9 | 37 | 36·2 | 0·45 | 1·20 | Urine |
| 9 | 290 | 287·9 | 1·09 | 0·38 | Plasma |
| 9 | 569 | 568·1 | 2·14 | 0·38 | Urine QC* |
| 9 | 1026 | 1026 | 3·53 | 0·34 | Urine QC† |

* Lyphocheck Quantitative urine control Normal (I), Lot No. 15300.

† Lyphocheck Quantitative urine control Abnormal (II), Lot No. 15400.

*Between-day precision*

The results are shown in table 2. The four levels of osmolality selected, representing clinically significant values, exhibit CVs of 0·34–1·20% (average 0·58%), which is satisfactory.

*Accuracy*

As shown in figure 1a, a split sample comparison between vapour pressure and freezing point measurements indicates good agreement. However, it is important to note that calibration of the Wescor vapour pressure osmometer is performed with aqueous standards having osmolality values established by our laboratory. For this reason, two additional accuracy studies were performed. First, a split sample comparison was made between the Advanced micro-osmometer and another freezing instrument (Knauer). As a result, a regression line of $y = 2{\cdot}5 - 1{\cdot}0x$ was calculated with a correlation coefficient of 0·9999. Second, osmolality values obtained with the Advanced micro-osmometer from samples were compared with values established by an external assessor, with satisfactory results (figure 1b).

*Linearity*

Table 3 indicates that the Advanced micro-osmometer gives a linear response up to at least 1917 mmol/kg. It is also interesting that the diluent used (distilled water, tube 1) has a value of 1 mmol/kg. Distilled water measurements with the vapour pressure osmometer resulted in osmolalities of 30–50 mmol/kg, indicating a possible deviation from linearity at low levels or contamination of the measuring chamber.

*Operator variation*

Previous experience had taught us to investigate this important, but often neglected variable. This becomes even more important if micro-amounts of sample are required by the analytical principle. Table 4 shows a CV range of 0·17–0·81% (average 0·49%) with accuracy deviations of less than 1% for all of the seven operators involved.

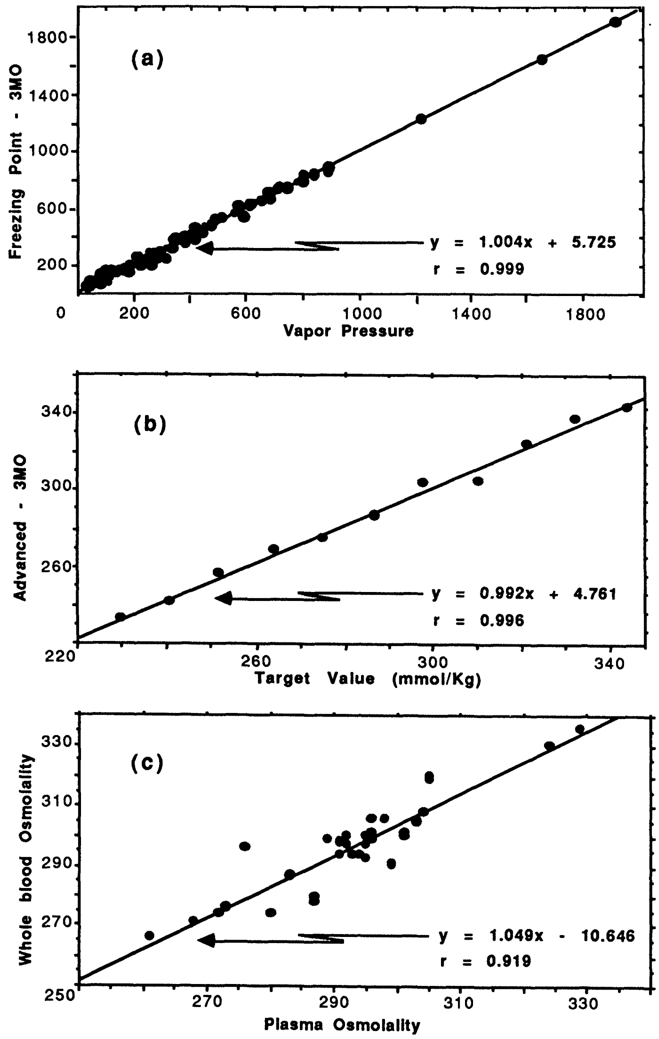

*Figure 1. Graphical representation of (a) split sample comparison of osmolality measurements by the Wescor vapour pressure and Advanced 3MO osmometers, (b) freezing point osmolality measurements of 11 external quality assurance samples and (c) whole-blood versus plasma osmolality measured by freezing point depression.*

*Table 3. Linearity*

| Tube | Sample (ml) | Diluent (ml) | Result* | Result* | Mean | Error (%) |
|---|---|---|---|---|---|---|
| 1 | 0·0 | 1·0 | 1 | 1 | 1 | – |
| 2 | 0·2 | 0·8 | 381 | 386 | 383·5 | −0·13 |
| 3 | 0·4 | 0·6 | 769 | 762 | 765·5 | −0·20 |
| 4 | 0·6 | 0·4 | 1140 | 1135 | 1138 | −1·00 |
| 5 | 0·8 | 0·2 | 1420 | 1530 | 1525 | −0·59 |
| 6 | 1·0 | 0·0 | 1904 | 1918 | 1911 | −0·31 |

*Regression analysis:*

| | |
|---|---|
| Slope | 0·9947 |
| Intercept | 0·41 |
| Correlation coefficient ($r$) | 0·9999 |

* Mean of duplicate measurements (mmol/kg).

*Table 4. Between-operator variation*

| Number of analyses | Target* (mmol/kg) | Mean (mmol/kg) | SD (mmol/kg) | CV (%) | Accuracy (%) | Operator type |
|---|---|---|---|---|---|---|
| 10 | 296 | 295·6 | 1·00 | 0·35 | −0·14 | Senior scientific |
| 10 | 296 | 295·2 | 0·92 | 0·31 | −0·27 | Senior technical |
| 10 | 279 | 277·7 | 1·77 | 0·64 | −0·47 | Senior scientific |
| 10 | 401 | 403·6 | 0·70 | 0·17 | +0·65 | Technical |
| 10 | 285 | 283·7 | 2·30 | 0·81 | −0·46 | Technical |
| 10 | 286 | 288·0 | 2·11 | 0·73 | +0·70 | Trainee scientific |
| 10 | 296 | 296·2 | 1·78 | 0·60 | +0·07 | Trainee technical |

* Target values established from previous measurements.

*Whole-blood osmolality*

Although a relatively small number of samples were analysed ($n = 34$) for whole-blood osmolality, the results shown in figure 1c indicate a high correlation between plasma and heparinized whole-blood samples. Note that in table 1 a CV of 1·02% was calculated, which compares with CVs of 0·17% at 290 mmol/kg and 0·35% at 295 mmol/kg for similar plasma measurements.

### Discussion

Our experience over the last 2 years with vapour pressure measurements of osmolality with all types of samples commonly submitted to a clinical laboratory (including sweat) has shown that this procedure lacks the precision and accuracy required by our laboratory, and this was confirmed by this study. We measured precision of our vapour pressure osmometer (unpublished results) and the CV was found to be of the order of 3·3–4·9% for an osmolality range of 74–1000 mmol/kg. Similar findings were reported by Davis [5] and Kaplan [6] in CAP quality assurance surveys and by the Royal College of Pathologists of Australasia in quality assurance surveys in the last 2 years [7,8]. This type of measurement also tends to produce lower results by an average of about 3% throughout the analytical range [5–8] which led us to alter the calibration values of primary standards in order to compensate for this effect.

The introduction of the Advanced micro-osmometer with a small sample requirement (20 µl) should enable the analyst to perform freezing point measurements with higher precision and accuracy than with earlier models of such instruments with larger sample size requirements.

This evaluation of the Advanced micro-osmometer has shown excellent precision and accuracy throughout the analytical range prescribed by the manufacturer. The CVs for within-run (table 1) and between-day (table 2) imprecision are well within acceptable limits using five different types of samples. It is linear throughout the analytical range, especially at low values, which will now enable us to measure sweat osmolality with confidence, whereas this was not possible with earlier models using the freezing point depression principle.

It is recognized that any analytical procedure is more or less susceptible to errors introduced by operator techniques and expertise. In this instance, we measured this important factor and the results shown in table 4 indicate a range of CVs between 0·17 and 0·81% with less than 0·7% deviation in analytical accuracy.

The high degree of precision exhibited by the Advanced micro-osmometer could be explained by examining the attempts made by the manufacturer to minimize factors that contribute to a loss of precision with conventional instruments. For example, bath temperature control, which is dependent on the fluid used, amount of fluid and volume changes as moisture condenses from the room air, may be important. The supercooling of the sample should be rapid but at a controlled rate. The Advanced instrument uses no fluid in the cooling chamber; instead, temperature is controlled by an electronically adjusted thermoelectric module that provides steady supercooling of the sample. The probe senses the temperature in the sample cell. Because the probe sees only a small portion of the sample space, it is important that the sample temperature be uniform. This is achieved by the standardized sample cuvettes which fit perfectly with the sample port. In addition, if the tip of the probe is not positioned repeatably from sample to sample, the precision may suffer. The tip of the probe in the Advanced instrument is in a fixed position and the sample port provides for repeatable positioning and centring of the sample in the probe. In most instruments the probe needs to be wiped in order to minimize carryover from sample to sample, and in laboratories with a large number of staff operating the instrument insufficient or incorrect cleaning could be encountered. Cleaning of the probe in the Advanced instrument is another regulated procedure which will ensure a clean chamber and probe each time with any operator.

Rocks *et al.* [9], using a freezing point osmometer, measured whole-blood osmolality and reported this to be an average of 1·5 mmol/kg higher than that of plasma. Certain factors must be considered if whole-blood osmolality is to be measured. These include effects of lithium heparin on whole-blood osmolality, effect of the packed cell volume and haemolysis. We did not investigate these effects in this work, but we are in the process of defining them in a much larger study. However, as it has been reported that the above factors may have no effect on osmolality measurements by freezing point depression [9], we have shown preliminary results in figure 1c.

Our results are in agreement with those of Rocks *et al.*, but we found whole-blood osmolality to be about 4.0 mmol/kg higher than plasma osmolality using lithium heparinized samples. It is believed that this is due to an artificial elevation caused by anaerobic glycolysis in the samples, which is unavoidable unless whole-blood osmolality is measured within 1 h of collection, and we intend to address this problem. Notwithstanding the above limitation, whole-blood osmolality could prove to be a very useful measurement, especially with subjects where sample size is of importance.

In conclusion, we have found the Advanced micro-osmometer to be a very precise and accurate instrument for the measurement of osmolality with a high degree of linearity throughout a wide analytical range, and the instrument design has almost entirely eliminated analytical errors introduced by operator techniques.

### Acknowledgements

The authors thank Stansens Scientific (Australia) for supplying the instrument and Miss Sandy Li for typing the manuscript.

### References

1. Webster, H. L. and Lochlin, H., *Medical Journal of Australia*, **1** (1977), 923.
2. Webster, H. L., and Barlow, W. K., *Clinical Chemistry*, **27** (1981), 385.
3. Webster, H. L., *CRC Critical Reviews in Clinical Laboratory Sciences*, **18** (1983), 313.
4. Carter, E. P., Barrett, A. D., Heeley, A. F., and Kuzemko, J. A., *Archives of Disease in Childhood*, **59** (1984), 919.
5. Davis, J. E., in *Clinical Chemistry, Theory, Analysis and Correlation*, Eds Kaplan, L. A., and Pesce, A. J. (C. V. Mosby, St. Louis, 1984), p. 237.
6. Kaplan, L. A., in Clinical Chemistry theory, analysis and correlation, Eds Kaplan, L. A., and Pesce, A. J. (C. V. Hosby, St. Louis, 1984), p. 1071.
7. Penberthy, L. A., *The Clinical Biochemist—Reviews*, **6** (1986), 146.
8. Penberthy, L. A., *The Clinical Biochemist—Reviews*, **8** (1987), 30.
9. Rocks, B. F., Sherwood, R. A., and Cook, J. G. H., *Annals of Clinical Biochemistry*, **23** (1986), 106.

